# Iron Administration Partially Ameliorates Cadmium-Induced Oxidative Damage in the Liver and Kidney of Rats

**DOI:** 10.1155/2024/6197553

**Published:** 2024-11-12

**Authors:** Ogechukwu E. Ezim, Lilian Kidi, Lauritta C. Ndufeiya-Kumasi, Sunny O. Abarikwu

**Affiliations:** Department of Biochemistry, University of Port Harcourt, Choba, Nigeria

**Keywords:** cadmium, concentration, iron, kidney, liver, oxidative stress

## Abstract

The protective effect of Fe against Cd-induced toxicity in the liver and kidney of rats during concurrent administration of both metals was investigated in this study. Fifty female rats (130–150 g) were distributed into five groups of 10 rats each (*n* = 10): Group I (control), received normal saline solution; Group II (1.2 mg CdCl_2_/kg b.w.); Group III (1.2 mg CdCl_2_ + 0.25 mg FeCl_2_/kg b.w.); Group IV (1.2 mg CdCl_2_ + 0.75 mg FeCl_2_/kg b.w.); and Group V (1.2 mg CdCl_2_ + 1.5 mg FeCl_2_/kg b.w.). Administration of both tested substances lasted for 47 days. Cd was injected intraperitoneally once a week, while Fe was administered to the Cd-exposed animals by oral gavage thrice weekly. The animals were killed at the end of the study, their blood was collected, and their liver and kidneys were harvested for biochemical and histological analysis. Following Cd administration, the kidney and liver showed a significant increase in Cd concentration, while Fe concentration in the kidney decreased. However, cotreatment with Fe decreased Cd concentration in the kidney and liver and increased Fe concentration in the kidney but not the liver, and the effect was more pronounced in the higher than lower doses. In the kidney, cotreatment with Fe especially at higher doses inhibited Cd-induced lipid peroxidation and plasma uric acid concentration. In the liver, lipid peroxidation which Cd did not alter was found to be elevated after cotreatment with the highest dose Fe. Inflammatory cell infiltrations of the central vein and renal tubular and glomeruli injury induced by Cd were not obviated by Fe cotreatment. It seems that both tissues respond differently to the concurrent administration of these metals and that Fe protected the kidney against oxidative injury-induced by Cd but not histopathological changes in both tissues.

## 1. Introduction

The increase in environmental contamination by toxicants and its adverse effect on human health has become an issue of growing concern around the globe [1]. Environmental toxicants consist of a wide range of chemical pollutants most of which are metals [2]. Within the last century, there have been massive increases in human exposure to heavy metals due to increasing industrial activities [3]. In developed and developing countries, the rate of food exposure to metals has increased due to the use of metallic contaminated fertilizers and the use of metallic contaminated water sources in agriculture. Thus, human exposure to one or more of these metals is nearly inevitable [4]. The most common heavy metals that cause human poisoning are mercury, lead, chromium, cadmium (Cd), and arsenic [3]. These heavy metals are released into the atmosphere from both natural sources and human activities especially during mining process [5, 6]. These emitted metals remain in the environment long after mining activities have ceased [6]. Rise in industrial and agricultural practices has led to increased Cd levels in the environment, and Cd is toxic even at very low concentrations [7–9]. The source of Cd intake in the general population is the diet [4], and most of the absorbed metal even at low concentrations accumulates in vulnerable tissues such as the kidneys and liver, contributing to several human health deficits including carcinogenesis, nephrotoxicity, and hepatotoxicity, becoming a global concern [7, 10, 11].

At the molecular level, Cd complexes with metallothionein (MT) and the thiol (SH)-containing antioxidant cellular component before it is transported through the glomeruli and reabsorbed by the renal tubules leading to tubular dysfunction [12]. Because MT is highly expressed in the hepatocytes of the liver, the Cd–MT complex also accumulates in the liver and causes hepatic toxicity [13]. The resulting hepatorenal toxicity has been associated with mitochondrial dysfunction, decrease in ATP, unfolding of proteins, direct DNA fragmentation, and finally induction of apoptotic pathways [13, 14]. Some studies have also confirmed that chronic exposure to even lower levels of Cd can result in early signs of renal toxicity and oxidative damage characterized by tubular proteinuria [15–17]. In the liver, Cd causes nonspecific inflammation, hepatocyte swelling, mild necrosis, and oxidative stress [18, 19]. It is believed that chronic or acute Cd exposures result in the deregulation of the homeostasis of essential minerals, e.g., Fe, through essential metal pathways to induce liver toxicity [14].

It is now established that mineral nutrition plays a regulatory role in heavy metal toxicity [20]. This is especially true of the essential micronutrients including zinc, copper, and iron (Fe) with respect to the toxicity of Cd. This is because Cd toxicity has been reported to be influenced by deficiency in Fe and zinc [3, 20–23]. Crowe and Morgan [24] reported that the accumulation of Cd in the growing rat liver was enhanced by Fe deficiency. This has also been found to be true in the liver and kidney of chicks that receive Fe-deficient diet [25]. Furthermore, Cd-induced Fe insufficiency is a major cause of liver and kidney damage in the rodent, bank vole, through the disturbance of Fe-dependent oxidative processes in both tissues [26]. Also, Fe deficiency was shown to increase duodenal mucosa Cd concentration and the deposition of the absorbed Cd in the kidney [21]. It therefore appears that Fe deficiency or insufficiency would enhance Cd toxicity and that Fe can protect against the toxicity of Cd in tissues [3, 26, 27]. The purpose of the present study is to evaluate if Fe has protective effects against Cd-induced hepatic and renal toxicity during concurrent exposure to both metals.

## 2. Materials and Methods

### 2.1. Chemicals and Reagents

Cadmium chloride (CdCl_2_) and ferrous sulfate (FeSO_4_) were purchased from Sigma-Aldrich (St. Louis, MO, USA). The chemicals were prepared weekly in normal saline (vehicle) and used throughout the study. The other reagents utilized in this study were of analytical grade and commercially available except were otherwise stated.

### 2.2. Animals and Treatment

Adult female Wistar (7–9 weeks old) rats weighing 130–150 g were purchased from the animal house of the Department of Biochemistry, University of Port Harcourt, and allowed to acclimatize for a week before the start of the experiment. Throughout the experiment, the animals had unrestricted access to rat chow and drinking water. Additionally, internationally recognized guidelines approved for this study by the Animal Research Ethics Committee of the Department of Biochemistry (UPH/BCHREC/2024/018) were followed throughout the period of the animal handling and sacrifice (National Institute of Health Publication Number, 85-23) [28]. The 50 female animals were randomly distributed into five groups of 10 animals per each group according to their body weights. The following treatment procedure was adopted for the study: Group I: Control received normal saline by gavage (2 mL/kg b.w.) for 47 days; Group II: Cd treated, received one dose of Cd (1.2 mg/kg b.w., intraperitoneally [*ip*]) once a week for 47 days for inducing tissue toxicity; Group III: Cd + Fe (small dose) treated, received Cd at 1.2 mg/kg b.w., *ip* once a week, and Fe at 0.25 mg/kg b.w., *p.o.* three times a week for 47 days; Group IV: Cd + Fe (medium dose) treated, received Cd at 1.2 mg/kg b.w., *ip* once a week, and Fe at 0.75 mg/kg b.w., *p.o.* three times a week for 47 days; and Group V: Cd + Fe (large dose) treated, received Cd at 1.2 mg/kg b.w., *ip* once a week, and Fe at 1.5 mg/kg b.w., *p.o.* three times a week for 47 days. The dose of 1.2 mg/kg *ip* is the lowest dose of Cd capable of inducing changes in rat's tissues and has been found to be more consistent with environmental contamination [29, 30]. Because toxicity begins at doses above 10–20 mg/kg b.w. of elemental Fe [31], the doses of Fe chosen in the present study were calculated from the therapeutic doses for Fe deficiency anemia, which is reported in the literature to be between 3 and 6 mg/kg/day [32, 33]. However, since the least dose (3 mg/kg b.w.) in these studies above elevated malondialdehyde (MDA) levels in the liver homogenates of rats from our pilot study, we chose doses lower than it which has no lipid peroxidation effect. Furthermore, our preliminary studies did not show any significant effect of Fe even at the highest studied dose (1.5 mg/kg b.w.) on most of the toxicity variables that were evaluated in this study (Supporting data, Tables 1 and 2). After 47 days, rats were fasted overnight and then anesthetized with chloroform in a closed chamber followed by decapitation for blood sample collection. The blood sample was collected into heparinized bottles until the animal stopped breathing and the blood stopped flowing. This duration of treatment was chosen to allow for the accumulation of Cd in the liver and kidney and to alter Fe homeostasis as reported previously for the gonadal tissues [27]. The livers and kidneys were excised, rinsed with phosphate-buffered saline (PBS, pH 7.4), pat dry in between Whatman paper, and weighed. Portions of each liver were cut and together with the left kidney were processed separately and immediately used for biochemical analysis of the oxidative stress variables, while the other portions of the liver and the right kidney were routinely processed for light microscopy. The plasma samples were centrifuged, and the supernatants obtained were processed for the estimation of aspartate aminotransferase (AST) activity and uric acid (UA) concentration.

#### 2.2.1. Sample Preparations for Biochemical Assays of Oxidative Stress Variables in Tissue Homogenates

The left kidney and portions of the liver were minced in 4 vol. of ice-cold 0.1 M phosphate buffer (pH 7.4) and homogenized using mortar and pestle placed on a tray of ice. The resulting samples were centrifuged at 10,000 revolutions per minute at 4°C for 30 min for the isolation of the postmitochondrial supernatant samples. The tissue supernatant samples were stored in the freezer until further biochemical analysis.

### 2.3. Evaluation of Cd and Fe Levels by Atomic Absorption Spectrophotometric Technique

Slices of the kidney and liver samples destined to be used for analyzing Cd and Fe concentrations were dried in an oven at 60°C for 24 h and then weighed. The dried tissues were added to an aqua regia solution (3:1 ratio of a mixture of concentrated HNO_3_ and HCl, respectively) and digested at 120°C until the fumes were clear and the solution was white. The digested tissues were mixed with deionized water, and the concentrations of Cd and Fe in the filtrate were analyzed using the flame atomic absorption spectrophotometry technique (Solar Thermo Elemental Spectrophotometer, Model SE-71906, Cd and Fe detection limits = 0.0001 ppm). The metal concentrations in the tissues were expressed as milligrams of metal per kilogram.

### 2.4. Determination of Concentration of Thiobarbituric Acid Reactive Substances (TBARS)−MDA Level in Liver and Kidney

Lipid peroxidation in the sample was evaluated by the spectrophotometric measurement of the level of MDA at 532 nm using thiobarbituric acid for color development [34]. The TBARS levels in the sample homogenates were measured as MDA, and the concentration of MDA was expressed as micromole per milligram protein. The results were calculated using an index of absorption for MDA of 1.56 × 10^5^/M/cm.

### 2.5. Estimation of Reduced Glutathione (GSH) Level and Activities of Antioxidant Defense Enzymes in the Liver and Kidney

Reduced GSH was evaluated according to the method of Sedlak and Lindsay [35] using 5, 5′-dithiobis-(2-nitrobenzoic acid) for color development. The absorbance was read in a spectrophotometer, and the results were expressed as *μ*g/mg protein. The activity of GSH *S-*transferase (GST) was measured in a spectrophotometer at 340 nm using 1-chloro-2, 4-dintrobenzene as a substrate [36]. The results were expressed as U/mg protein. Superoxide dismutase (SOD) was estimated as described previously by Kakkar, Das, and Viswanathan [37]. Briefly, the sample (50 *μ*L) was mixed with 650 *μ*L of sodium pyrophosphate buffer (pH 8.3, 0.052 M), 50 *μ*L of phenazine methosulfate (186 *μ*M), 150 *μ*L of nitroblue tetrazolium chloride (300 *μ*M), and 100 *μ*L of NADH (780 *μ*M) and was vortexed thoroughly. After 90 s of incubation, 500 *μ*L of glacial acetic acid was added to stop the reaction. The reaction mixture was kept at room temperature for 10 min. The absorbance of the reaction mixture was measured at 560 nm, and the results were expressed as U/mg protein. Catalase (CAT) assay was measured spectrophotometrically at 240 nm by the method of Chance and Maehly [38]. Briefly, 2.5 mL of phosphate buffer (pH = 5.4, 0.1 M) was mixed with 0.4 mL of 5.9 mM H_2_O_2_ and 0.1 mL of sample in a cuvette. The reaction mixture was mixed by inversion and allowed to equilibrate for 1 min in a prewarmed spectrophotometer. The change in absorbance was read at 240 nm at 10-s intervals for 1 min. One unit of CAT activity equals to the amount of protein that converts 1 µmoL H_2_O_2_ per minute to H_2_O and O_2_. The activity was expressed as U/mg protein. GSH peroxidase (GSH-Px) assay was quantified based on the measurement of the GSH used up after the samples were mixed with the substrates, H_2_O_2_ (2.5 mM) and GSH (4 mM). The absorbance was read on a spectrophotometer at 412 nm against a blank containing only phosphate solution and Ellman's reagent. The concentration of GSH was extrapolated from a GSH standard curve (0–1000 *μ*M), and the enzymatic activity was expressed as *μ*g GSH consumed/min/mg protein [39]. Protein concentrations in the samples were determined as described by Lowry et al. [40].

### 2.6. Evaluation of Biochemical Markers of Hepatic and Renal Functions

The activity of AST and UA levels was assayed in the plasma samples using the Randox commercial kits (RANDOX Laboratories Ltd, Crumlin, UK) following the instructions provided by the manufacturers.

### 2.7. Histopathological Observations of the Liver and Kidney

The isolated right kidney from each animal and a portion of the liver were fixed in 10% buffered formalin for 24 h. The tissues were dehydrated through an alcohol-graded series and finally embedded in paraffin wax according to routine procedure. At least five slides were prepared from each animal, with tissue sections of 5 *μ*m thickness. The sections were stained with hematoxylin and eosin and observed under a light microscope (L × microscope, Labomed, USA) for histological changes.

### 2.8. Statistical Analysis

Data were expressed as mean ± standard error of mean. Data sets were found to be normally distributed around the mean when tested for normality using the Shapiro–Wilk test. Furthermore, the equality of variances assumption was tested by Levene's test and was found to be homogenous across data sets. All statistical calculations were done with one-way ANOVA using GraphPad Prism 6 (GraphPad Software, Inc., San Diego, CA, USA). When statistically significant differences were found, Tukey's post hoc multiple comparison test was used to analyze the differences of means. Significant difference was set at *p* < 0.05.

## 3. Results and Discussion

### 3.1. Effect of Cd and Fe on Oxidative Stress Variables in the Kidney and Liver Homogenates of Rats

Treatment with Cd increased the level of TBARS and decreased GSH concentration and SOD, CAT, GSH-Px, and GST activities in the kidney of the Cd group compared with the control (Table 1) (*p* < 0.05). A significant increase (*p* < 0.05) in the level of GSH was observed in the Cd + large-dose Fe and Cd + medium-dose Fe groups compared to the Cd group. The increase in SOD activity in the combined exposure group was higher in the large-dose Fe group than the lower doses, but the increase did not reach significant levels in the Cd + large-dose Fe group versus the control values (*p* > 0.05). Treatment with Cd + small-dose Fe, Cd + medium-dose Fe, and Cd + large-dose Fe significantly increases (*p* < 0.05) CAT, GSH-Px, and GST activities compared to the Cd group. The increase in the GST activity was more conspicuous in the Cd + small-dose Fe compared to the other combined exposure groups, but the increase did not reach statistically significant values (*p* > 0.05).

Table 2 shows the TBARS level and the antioxidant status in the liver of the experimental animals. TBARS measured as MDA level remained relatively insignificant after Cd treatment and on cotreatment with the lowest- and medium-dose Fe compared with the control (*p* > 0.05). There was a tendency for MDA concentration to increase on cotreatment with the largest dose Fe which reached a significant difference compared to the control and other treatment groups (*p* < 0.05). Furthermore, Cd treatment decreased GSH concentration which was prevented on cotreatment with lowest and medium-dose Fe but not with the highest Fe dose. Additionally, the activity of SOD showed a significant decrease (*p* < 0.05) only in the Cd + large-dose Fe group compared to the other groups, and remained relatively unaffected in the other groups compared to the control values (*p* > 0.05). Treatment with Cd significantly increased CAT activity in the liver compared with the control, and the observed decrease in the combined exposure groups became significantly different from the control value in the Cd + large-dose Fe-treated animals compared with the control. In addition, treatment with Cd alone and on cotreatment with Fe at the different tested doses did not significantly change GSH-Px activity compared to the control (*p* < 0.05). There was a significant increase in GST activity in the liver of Cd-treated animals and also in animals cotreated with Fe at the different doses when compared to the control (*p* < 0.05). The elevated CAT and GST activities and the unchanged TBARS level in the liver confirm that the hepatic system has robust antioxidant defenses that protect it against oxidative damage [41, 42]. Furthermore, Fe administration at small and medium doses following Cd intake inhibited Cd-induced fluctuations in GSH concentration and CAT activity and did not change MDA concentration. However, MDA levels remained high when concurrent administration of the large-dose Fe was followed by Cd intake. This is assumed to be due to the decreased hepatic GSH level as well as the inhibited CAT and SOD activities induced by the large-dose Fe in these animals [42]. Although it is shown that endogenous defense mechanisms largely prevent TBARS formation in the liver, the presence of Fe in hepatic tissues can promote oxidative damage [43], thereby supporting our findings in this study. Other studies have also established that the liver is the main organ affected by the oxidative stress caused by Fe toxicity [44, 45]. In the liver, Fe undergoes redox cycling in the presence of superoxide radicals (O_2_•^−^) and H_2_O_2_ through the Haber–Weiss and Fenton reactions to generate the short-lived and highly reactive hydroxyl radicals (OH•). The resulting OH• is a potent initiator for phospholipid peroxidation and oxidative cellular damage [45, 46] and may also give rise to other reactive free radicals such as alkoxy radicals to propagate the chain reaction of lipid peroxidation [43]. This suggests that Fe may have varying effects depending on the dose and affected tissue, and might not always acts as a uniform protector against Cd toxicity.

Because the Cd-induced GST activity remained unabated in the different Fe cotreatment groups could be interpreted to mean that its activity in the liver of Cd-treated animals is responsible for the decreased hepatic GSH concentration [47], it is assumed that the elevated hepatic GST activity caused by Cd was not a protective response to oxidative stress since the hepatic tissues of the animals in the large-dose Fe cotreatment group also have elevated TBARS concentrations [42]. On the other hand, the fluctuations in these oxidative stress indicators in the kidney are in accordance with the literature on Cd-induced nephrotoxicity associated with oxidative stress. For instance, the decreased CAT, SOD, and GSH-Px activities and GSH concentration have been observed in the kidney of rats exposed to Cd [11] at a dose similar to the one used in the present study, thereby corroborating the present findings.

Furthermore, several experimental animal models including rats, rabbits, and mice usually respond to Cd treatment by decreasing GST enzyme activity levels in renal tissues [48]. It is important to note that GSH is a component of the GSH-Px and GST detoxification system for the inactivation of metals and removal of active oxygen free radicals [48]. In tissues, Cd binds GSH with high affinity making it unavailable [48]. It is therefore speculated that the decrease in GST and GSH-Px activities in the kidney may be a consequence of the depleting effect of Cd on GSH concentration resulting to the oxidative stress. Many other studies have also established the decrease in enzymatic and nonenzymatic antioxidant systems and the triggering of oxidative stress as important components of Cd-induced renal injury [49–51]. Furthermore, some studies found that CAT activity was decreased in the hepatic and renal tissues of rats exposed to Cd [11, 52, 53]. In some of these studies [11, 52, 53], Cd was administered at higher doses and for shorter duration than was applied in the present study. These differences in doses and duration of Cd treatment employed in the present study could be part of the reason for the observed increase in CAT activity in the liver of the Cd-treated animals. Notwithstanding, other studies with female rats support our present findings. For example, Pillai and Gupta [54] speculated that the increase in CAT activity in the liver of female rats treated with Cd is based on the fact that the antioxidant enzymes are synthesized in response to oxidative stress. Despite the Cd-induced changes in the antioxidant markers in the liver, the hepatic system appears to have a much more flexible antioxidant defense system that resisted hepatic lipid peroxidation [42]. Similarly, the results for the increased TBARS concentration in the kidney agree with the reports of other investigators [11, 55], an observation that is reported to be related to the accumulation of Cd in this tissue [55]. In the proximal tubule, Cd forms a complex with MT that is degraded in the lysosome to give rise to the unbound Cd that induces oxidative damage in renal tissues [12]. Nevertheless, concurrent administration of Cd with medium and large doses of Fe decreased TBARS concentration but not when the cotreatment was tested with the lowest dose of Fe (0.25 mg/kg b.w.), suggesting that at low doses, Fe may not effectively prevent Cd-induced oxidative damage. Apparently, tissue differences contribute to the Cd-induced toxic responses in the biologic system, and the liver appears less susceptible than the kidney to Cd-induced oxidative damage since TBARS and the antioxidant markers, SOD and GSH-Px, remained unchanged in the liver following Cd treatment in this study. Other investigators have also established that Cd treatment decreased renal SOD activity [56]. It is believed that Cd interacts with the metal moieties of SOD such as copper, zinc, or manganese to lower its activity or that Cd may interact with the enzyme directly, change the conformation of the enzyme, and impair its functional activity [57]. However, low- and medium-dose Fe cotreatments decreased Cd-induced increase in CAT activity and increased Cd-induced decrease in GSH hepatic concentration, suggesting a better antioxidant effect of Fe at lower doses than at higher doses in the liver of Cd-cotreated rats. In the kidney, Fe cotreatment at all the tested doses in this study prevented Cd-induced deficits in the antioxidative parameters except for the SOD activity that was increased in the Cd + large-dose Fe group to levels that did not significantly reach the control values. This is due to the fact that the kidney accumulated Fe much more than the liver to the levels that elicited antioxidant protective effects against Cd-induced oxidative stress [26]. To support this assumption, the threshold level for tissue Cd required to induce morphological deficits has already been suggested [24, 58].

### 3.2. Effect of Cd and Fe on the Levels of the Metals in the Kidney and Liver Homogenates and Plasma Metals, UA Concentrations, and AST Activity

The concentrations of Cd and Fe in the kidney homogenates and plasma levels of UA in the experimental animals are shown in Table 3. Treatment with Cd significantly increased the plasma level of UA (*p* < 0.05). The increase in UA concentrations noted in the Cd-treated animals were significantly decreased toward control values in the Cd + medium-dose Fe and Cd + large-dose Fe but not Cd + small-dose Fe. As expected, exposure to Cd significantly increased the concentration of Cd in the kidney. This increase was significantly inhibited in the Cd + large-dose Fe and Cd + medium-dose Fe but not in the Cd + small-dose Fe compared to the Cd group. Furthermore, treatment with Cd significantly decreased the level of Fe in the kidney compared with the control, while an increase in the Fe level was observed in the combined exposure group which reached significant level beginning with the Cd + medium-dose Fe group compared to the Cd values (*p* < 0.05).

Table 4 shows the metal concentration in the liver and the plasma activity of AST, a marker of hepatic function. Treatment with Cd separately or in combination with Fe at the different doses resulted to a significant increase (*p* < 0.05) in AST activity compared to the control. As expected, Cd treatment alone resulted to a significant increase (*p* < 0.05) in the concentration of the metal in the liver followed by its decrease on cotreatment with Fe when compared with Cd values (*p* < 0.05). The large-dose Fe has more blunting effect on Cd level than the lower doses. The concentration of hepatic Fe concentration remained statistically unchanged across all groups when compared to with control (*p* > 0.05).  Table 5 shows the plasma levels of Cd and Fe in the experimental animals. Rats treated with Cd showed a significant decrease in plasma Fe levels compared to the control. As expected, there was a significant increase in Fe concentration on cotreatment with the different doses of Fe compared with Cd values (*p* < 0.05), and the increase was higher in the Cd + large-dose Fe than the lower dose exposure group. Similarly, Cd administration significantly increased the plasma Cd concentration compared with the control (*p* < 0.05), and the decrease in plasma Cd concentration on cotreatment with the different doses of Fe reached a statistically significant level compared to Cd values (*p* > 0.05).

Several studies have established the role Fe deficiency plays in Cd-induced liver and kidney toxicities in animal models [18, 59, 60]. It is known that Cd induces the metabolism of mineral nutrients including Fe in different body tissues [27, 61, 62]. The decrease in Fe concentration observed in the kidney of rats in this study is in agreement with the report of Jurczuk et al. [42] and is related to the fact that Fe homeostasis is impaired by Cd in the kidney [59]. This seems probable because Cd competes with Fe for entry in renal tissues apart from the other entry routes for Cd [63]. The unchanged hepatic Fe concentration following concurrent Cd treatment may then be attributed to differences in the hepatic handling of Cd which is influenced by the dosage, duration, and route of Cd administration. For instance, Djukic-Cosic et al. [60] found that hepatic Fe concentrations were decreased following oral gavage of Cd at 10 mg/kg b.w. This was also found to be true in nonhepatic tissues, even at higher doses of Cd [27] than was reported by Djukic-Cosic et al. [60]. It is therefore rational to assume that the 1.2 mg/kg b.w. dosage of Cd used in the present study was insufficient to induce the depletion of hepatic Fe level but not in the kidney, therefore confirming the susceptibility of the kidney more than the liver to Cd-induced toxicity [64]. Notwithstanding, when Cd was coadministered with the different tested doses of Fe, a decrease in Cd concentration was observed in the kidney and liver and an increase in Fe concentration in the kidney when compared to its concentrations in the Cd-only administered rats, suggesting that exogenous low-dose Fe is effective to prevent Cd accumulation in the liver and kidney under concurrently exposed experimental situation. Nevertheless, there seems to be a dosage limit for Fe preventive capability on Cd accumulation in susceptible tissues because Cd may inhibit Fe absorption at low to normal level of Fe, but at largely elevated level, Cd and Fe are transported into tissues by other noncompetitive mechanisms [24, 65]. In our previous studies with the gonadal system of mice, higher doses of both metals than those applied in this study have synergistic toxic effects and caused the accumulation of both metals in the testis, which is not the same observation reported here with much more smaller doses of Fe [65]. Thus, it is significant to consider the dose and context when evaluating Fe efficacy in future studies of Cd model of toxicity.

The elevated UA concentration and AST activity in the plasma of Cd-treated rats have also been confirmed in previous studies [66]. It is widely assumed that pathological alterations in the liver are indicated by high increases in plasma levels of AST [67]. Similarly, increases in plasma UA concentrations are indications of kidney dysfunctions which may result from damage to the kidney induced by the accumulation of Cd in renal tissues. Furthermore, elevated liver function markers may be due to necrosis of hepatocytes following chemical toxicity leading to increase in membrane damage and, consequently, the uncontrolled release of the liver enzymes into the blood [68]. Thus, the increase in plasma AST enzyme activity in the present study is due to damage of hepatic tissue and the resulting release of this enzyme into the blood circulation. The decrease in UA concentration in the plasma following medium-dose Fe and large-dose Fe cotreatments to levels comparable with the control confirms the protective effect of Fe against Cd-induced biochemical changes in the kidney of rats.

### 3.3. Effect of Cd and Fe on Renal and Liver Morphology

**Figure 1:**
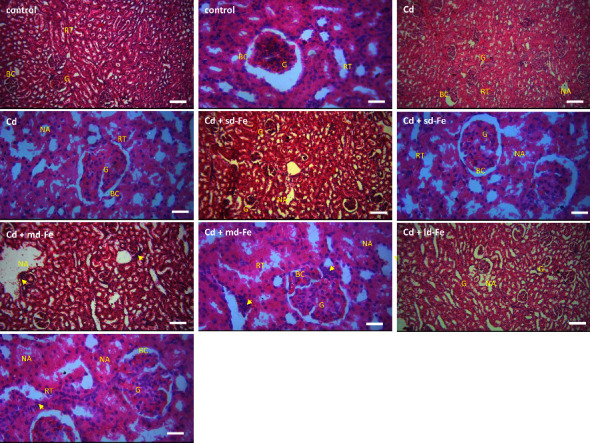
Representative photographs of the histological sections of the kidney from control rats and those exposed to cadmium (Cd) alone and when combined with different doses of iron (Fe). The glomeruli (G), renal tubules (RT), and Bowman's capsular space (BC) from control animals are preserved. Note the histologically distorted kidney in the Cd and Fe-cotreated groups showing necrotic area (NA), renal tubular destruction (RT), patchy areas of glomerular destruction (G), and interstitial inflammation (short arrows). Cd + sd-Fe (small-dose Fe), Cd + md-Fe (medium-dose iron), Cd + ld-Fe (large-dose iron). Hematoxylin and eosin stain (Mag. 100x., scale bar = 100 *μ*m; Mag. 400x., scale bar = 50 *μ*m).

Hematoxylin- and eosin-stained kidney tissue sections from rats in each group are shown in Figure 1. Kidney tissues from the control animals showed normal glomerular and tubular structures including glomerular mesangial cells, mesangial matrix and capillaries, interstitial cells, patent Bowman's capsular space, and renal tubules lined with simple epithelial cells. Cd-treated rats demonstrated necrotic areas (NA), renal tubular destruction (RT), and patchy areas of glomerular destruction (G). Small-dose iron (sd-Fe) cotreatment demonstrated histologically distorted kidney with lobulated glomeruli and NA. Similarly, medium-dose iron (md-Fe) showed histologically distorted kidney, NA, and inflammatory cells, whereas large-dose iron (ld-Fe) cotreatment showed histologically distorted kidney with globulated and shrunken glomeruli and NA.

**Figure 2:**
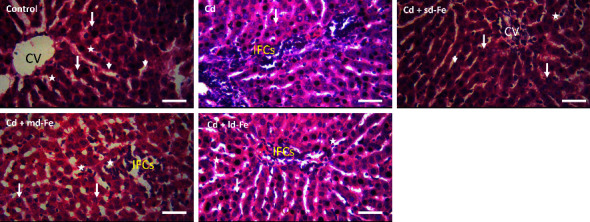
Representative microscopic images of the liver from control rats and those exposed to cadmium (Cd) alone and when combined with different doses of iron (Fe). In control, normal structure of the hepatocytes (long arrows) around the central vein (CV) is observed, and the presence of normal sinusoids (arrow heads) separating the interconnecting cords of hepatocytes and containing Kupffer cells (starred). In Cd, many mass of accumulated inflammatory cells (IFCs) around the central vein and many hepatocytes (arrows) that appeared normal were observed. In Cd + sd-Fe (small-dose Fe) cotreated animals, the hepatocytes appeared normal as well as the liver sinusoids separating the interconnecting cords of hepatocytes and also contain inflammatory cells (starred). However, the central veins (CV) are filled with cellular debris and inflammatory cells. In Cd + md-Fe (medium-dose iron) cotreated animals, the central veins that are surrounded by interconnecting cords of normal hepatocytes (arrows) and the sinusoids separating the interconnecting cords of hepatocytes showed aggregation of inflammatory cells (IFCs). In Cd + ld-Fe (large-dose iron) cotreatment, the cords of normal hepatocytes (arrows) radiating from the central veins showed slight aggregation of inflammatory cells and the presence of Kupffer cells in the normal sinusoids separating the interconnecting cords of hepatocytes. Hematoxylin and eosin stain (Mag. 400x, scale bar = 50 *μ*m).

Hematoxylin- and eosin-stained liver tissue sections from rats in each group are shown in Figure 2. Liver sections of the control animals showed normal hepatic lobule consisting of hepatocytes arranged in interconnecting cords in a radiating manner around the central veins. There is also the presence of normal sinusoids separating the interconnecting cords of hepatocytes and containing the Von Kupffer cells. After Cd treatment, many masses of accumulated inflammatory cells were found around the central vein. The hepatocytes also appeared to be normal in this group of animals. Small-dose iron (sd-Fe) cotreatment also showed histologically normal hepatic lobule consisting of hepatocytes arranged in interconnecting cords in a radiating manner around the central veins. There is also the presence of normal liver sinusoids separating the interconnecting cords of hepatocytes. However, the central veins were filled with inflammatory cells and debris and the sinusoids with inflammatory cells. The central veins that are surrounded by interconnecting cords of hepatocytes and the sinusoids showed aggregation of inflammatory cells following medium-dose iron (md-Fe) cotreatment. Large-dose iron (ld-Fe) cotreatment also showed aggregation of inflammatory cells and cords of normal hepatocytes radiating from the central vein. There is also the presence of normal sinusoids separating the interconnecting cords of hepatocytes and containing Von Kupffer cells. The histologic changes observed in the tissues of Cd-treated animals might not be connected to the oxidative damage [27, 58, 69]. It is assumed that lipid peroxidation and oxidative stress as reflected by the high MDA level and the fluctuated antioxidant systems are not always responsible for pathological changes induced by Cd in tissues [70]. Furthermore, biochemical and histological changes elicited by Cd and other toxic metals are not always linked [58]. There are therefore several mechanisms of Cd toxicity including direct damage to tissues which could not be obviated by Fe treatment in the present study. These might have contributed to the inability of exogenous Fe to preserve the histologic features of both tissues even though it prevented Cd-induced biochemical alterations. Furthermore, TBARS levels and the production of reactive oxygen species are the molecular features of Fe-related toxicity [71] and an important contributor of liver damage by Fe. The ld-Fe could promote inflammation and fibrosis, and the resulting hepatocyte necrosis is thought to be the real driving force leading to the fibrosis and the progression of various liver diseases [71, 72]. This is the first study to report that Fe does not have broad-based protective effects in a Cd model of hepato- and nephrotoxicity.

## 4. Conclusion

This study demonstrates that the liver has a much more robust flexible antioxidant system than the kidney that allowed limited oxidative damage despite the altered antioxidant defense induced by Cd. We further showed that Fe cotreatment inhibited the accumulation of Cd in renal tissue and diminished Cd-induced oxidative stress, highlighting that mineral nutrition management with Fe plays a role in reducing Cd toxicity. Overall, Fe intake that is equivalent to the human daily requirement is sufficient to protect the kidney from Cd-induced biochemical aberrations but might not be protective against all pathological changes induced by Cd in renal and hepatic tissues. These results provide a basis for developing more effective prevention and treatment strategies with Fe in future studies, aiming to prevent Cd intoxication and its effects.

## Figures and Tables

**Table 1:** Effect of Cd and cotreatment with Fe on oxidative stress variables in the kidney homogenates of rats.

Variables	Control	Cd	Cd + small-dose Fe	Cd + medium-dose Fe	Cd + large-dose Fe
TBARS (*μ*mol MDA/mg)	4.89 ± 0.82^a^	13.65 ± 3.53^b^	13.45 ± 2.53^b^	5.61 ± 2.46^c^	5.68 ± 0.58^c^
GSH (*μ*g GSH/mg)	663 ± 39^a^	542 ± 37^b^	583 ± 34^b^	603 ± 51^c^	638 ± 58^c^
SOD (U/mg)	4.43 ± 0.71^a^	3.47 ± 1.02^b^	3.18 ± 0.27^b^	2.97 ± 0.41^b^	3.84 ± 1.17^b^
CAT (U/min/mg)	297.15 ± 24^a^	260.44 ± 17^b^	399.75 ± 29^c^	396.74 ± 11.52^c^	345.89 ± 41.06^c^
GSH-Px (*μ*g GSH/mg)	306.6 ± 10.3^a^	271.8 ± 6.71^b^	325.9 ± 2.6^c^	307.3 ± 2.07^d^	287.7 ± 5.93^e^
GST (U/mg)	0.97 ± 0.25^a^	0.47 ± 0.09^b^	1.29 ± 0.39^a^	0.9 ± 0.16^a^	0.91 ± 0.053^a^

*Note*: Data are presented as mean ± standard deviation (number of samples used for statistical analysis = 10). Rows with different alphabets are significantly different (*p* < 0.05).

Abbreviations: CAT, catalase; GSH, reduced glutathione; GSH-Px, glutathione peroxidase; GST, glutathione S*-*transferase; MDA, malondialdehyde; SOD, superoxide dismutase; TBARS, thiobarbituric acid reactive substances.

**Table 2:** Effect of Cd and cotreatment with Fe on oxidative stress variables in the liver homogenates of rats.

Variables	Control	Cd	Cd + small-dose Fe	Cd + medium-dose Fe	Cd + large-dose Fe
TBARS (*μ*mol MDA/mg)	21.10 ± 6.37^a^	18.06 ± 1.87^a^	19.94 ± 5.93^a^	19.33 ± 3.15^a^	27.72 ± 2.34^ab^
GSH (U/mg)	1.59 ± 0.23^a^	1.32 ± 0.22^b^	2.09 ± 024^c^	1.82 ± 0.23^c^	1.20 ± 0.06^b^
SOD (U/mg)	3.14 ± 0.41^a^	2.97 ± 0.34^a^	2.75 ± 0.42^a^	2.57 ± 0.48^a^	1.97 ± 0.22^b^
CAT (U/min/mg)	11.64 ± 3.12^a^	29.21 ± 0.93^b^	16.19 ± 1.73^a^	12.06 ± 1.44^a^	5.93 ± 1.85^c^
GSH-Px (*μ*g GSH/mg)	366.58 ± 4.6^a^	365.8 ± 4^a^	362.02 ± 5.13^a^	361.02 ± 5.39^a^	365.56 ± 6^a^
GST (U/mg)	4.69 ± 0.42^a^	7.75 ± 0.29^b^	8.87 ± 0.74^b^	8.1 ± 0.29^c^	7.45 ± 0.8^c^

*Note*: Data are presented as mean ± standard deviation (number of samples used for statistical analysis = 10). Rows with different alphabets are significantly different (*p* < 0.05).

Abbreviations: CAT, catalase; GSH, reduced glutathione; GSH-Px, glutathione peroxidase; GST, glutathione S*-*transferase; MDA, malondialdehyde; SOD, superoxide dismutase; TBARS, thiobarbituric acid reactive substances.

**Table 3:** Effect of Cd and cotreatment with Fe on plasma uric acid concentrations, Cd, and Fe contents in the kidneys of rats.

Variables	Control	Cd	Cd + small-dose Fe	Cd + medium-dose Fe	Cd + large-dose Fe
Uric acid (*μ*mol/L)	587 ± 173^a^	880 ± 142^b^	822 ± 105^b^	595 ± 101^a^	525 ± 28^a^
Cd (ppm)	0.23 ± 0.02^a^	0.28 ± 0.017^b^	0.21 ± 0.017^a^	0.18 ± 0.023^c^	0.13 ± 0.06^d^
Fe (ppm)	4.34 ± 0.61^a^	2.78 ± 0.32^b^	3.52 ± 0.22^a^	4.19 ± 0.33^c^	4.75 ± 0.32^d^

*Note*: Data are presented as mean ± standard deviation (number of samples used for statistical analysis = 10). Rows with different alphabets are significantly different (*p* < 0.05).

Abbreviations: Cd, cadmium; Fe, iron.

**Table 4:** Effect of Cd and cotreatment with Fe on plasma aspartate aminotransferase (AST) activity, Cd, and Fe contents in the liver of rats.

Variables	Control	Cd	Cd + small-dose Fe	Cd + medium-dose Fe	Cd + large-dose Fe
AST (U/l)	121 ± 22^a^	165 ± 26^b^	161 ± 13^b^	147 ± 25^ab^	140 ± 29^ab^
Cd (ppm)	0.23 ± 0.02^a^	0.38 ± 0.01^b^	0.33 ± 0.03^ab^	0.26 ± 0.03^d^	0.24 ± 0.06^c^
Fe (ppm)	1.91 ± 0.24^a^	2.18 ± 0.09^a^	1.79 ± 0.49^a^	2.15 ± 0.18^a^	2.05 ± 0.21^a^

*Note*: Data are presented as mean ± standard deviation (number of samples used for statistical analysis = 10). Rows with different alphabets are significantly different (*p* < 0.05).

Abbreviations: AST, aspartate aminotransferase; Cd, cadmium; Fe, iron.

**Table 5:** Effect of Cd and cotreatment with Fe on plasma Cd and Fe concentrations in rats.

Variables	Control	Cd	Cd + small-dose Fe	Cd + medium-dose Fe	Cd + large-dose Fe
Cd (ppm)	0.35 ± 0.07^a^	0.57 ± 0.08^b^	0.41 ± 0.13^a^	0.32 ± 0.07^c^	0.21 ± 0.07^d^
Fe (ppm)	3.69 ± 0.62^a^	0.23 ± 0.05^b^	2.31 ± 0.16^c^	3.26 ± 0.44^d^	3.42 ± 0.23^d^

*Note*: Data are presented as mean ± standard deviation (number of samples used for statistical analysis = 10). Rows with different alphabets are significantly different (*p* < 0.05).

Abbreviations: Cd, cadmium; Fe, iron.

## Data Availability

Data are available on request.

## Supporting Information

Intermittent administration of Fe at small dose (0.25 mg/kg b.w.), medium dose (0.75 mg/kg b.w.), and large dose (1.5 mg/kg b.w.) to rats 3 times a week by oral gavage did not change the oxidative stress variables: TBARS, GSH, SOD, GSH-Px, and GST in both the liver and kidney homogenates of rats after 47 days. Uric acid and AST used as markers for kidney injury and hepatotoxicity were also evaluated in the plasma and found not to change after 47 days, suggesting that the doses of Fe used in the present study had no acute toxicity effect in rats (Supporting Tables 1 and 2).
