# Supplementary material for: Iron Administration Partially Ameliorates Cadmium-Induced Oxidative Damage in the Liver and Kidney of Rats
Source: J Toxicol. 2024 Nov 12;2024:6197553. doi: 10.1155/2024/6197553 (PMC11576088; doi:10.1155/2024/6197553)
Supplement: Supporting Information — Additional supporting information can be found online in the Supporting Information section. [file 6197553.f1.doc]

Supplementary Table 1: Effect of intermittent Fe supplementation on oxidative stress variables in the kidney homogenates and uric acid level in the plasma of rats after 47 days.

Variables control small dose Fe medium dose Fe large dose Fe

TBARS (µmol mda/mg) 5.17 ± 1.26 5.62 ± 2.14 6.19 ± 1.12 5.74 ± 2.11

GSH (µg GSH/mg) 634.19 ± 41 598.21 ± 37 643.46 ± 46 654.17 ± 32

SOD (U/mg) 4.21 ± 0.21 3.98 ± 0.42 4.19 ± 0.37 4.36 ± 0.68

GSH-Px (µg GSH/mg) 321 ± 21.43 329 ± 17.13 330 ± 14.32 337 ± 22.16

GST (U/mg) 1.2 ± 0.15 1.31 ± 0.36 1.37 ± 0.26 1.36 ± 0.53

Uric acid (µmol/l) 562 ± 135 539± 115 581 ± 102 537 ± 76

Thiobarbituric acid reactive substances (TBARS); reduced glutathione (GSH); superoxide dismutase (SOD); catalase (CAT); glutathione peroxidase (GSH-Px); glutathione *S-*transferase (GST); MDA = malondialdehyde; Data are not significantly different across groups for each variable (p > 0.05)

Supplementary Table 2: Effect of intermittent Fe supplementation on oxidative stress variables in the liver homogenates and AST activity in the plasma of rats after 47 days.

Variables control small dose Fe medium dose Fe large dose Fe

TBARS (µmol mda/mg) 24.12 ± 5.43 21.42 ± 9.07 25.31 ± 5.73 26.72 ± 3.72

GSH (µg GSH/mg) 2.17 ± 0.74 2.36 ± 0.62 2.18 ± 0.52 2.09 ± 0.65

SOD (U/mg) 3.26 ± 0.39 2.93 ± 0.71 2.86 ± 0.57 2.89 ± 0.81

GSH-Px (µg GSH/mg) 386 ± 10.75 381 ± 25.32 379 ± 23.12 384 ± 17.63

GST (U/mg) 5.39 ± 0.52 6.34 ± 0.79 6.19 ± 0.62 6.45 ± 0.89

AST (µmol/l) 127.35 ± 27 131.14 ± 28 134.72 ± 24.75 132.15 ± 29.61

Thiobarbituric acid reactive substances (TBARS); reduced glutathione (GSH); superoxide dismutase (SOD); catalase (CAT); glutathione peroxidase (GSH-Px); glutathione *S-*transferase (GST); MDA = malondialdehyde; AST = aspartate aminotransferase; Data are not significantly different across groups for each variable (p > 0.05)
